# Immediate breast reconstruction for women having inflammatory breast cancer in the United States

**DOI:** 10.1002/cam4.1546

**Published:** 2018-05-15

**Authors:** Sameer A. Patel, Marilyn Ng, Salvatore M. Nardello, Karen Ruth, Richard J. Bleicher

**Affiliations:** ^1^ Department of Surgical Oncology Fox Chase Cancer Center Philadelphia PA USA; ^2^ Division Plastic, Reconstructive and Hand Surgery Department of Surgery Northwell Health‐Staten Island University Hospital Staten Island NY USA; ^3^ Department of Surgery Hallmark Health System Stoneham MA USA; ^4^ Department of Biostatistics Fox Chase Cancer Center Philadelphia PA USA

**Keywords:** breast neoplasms, inflammatory breast neoplasms, patient safety, practice guideline, reconstructive surgical procedures

## Abstract

Inflammatory breast cancer (IBC) is an aggressive malignancy having a poor prognosis. Traditionally, reconstruction is not offered due to concerns about treatment delay, margin positivity, recurrence, and poor long‐term survival. There is a paucity of literature, however, evaluating whether immediate breast reconstruction (IBR) is associated with greater mortality in patients with IBC. A population‐based study was conducted via the SEER‐Medicare‐linked database (1991‐2009). Female patients greater than 65 years were reviewed who had mastectomy and reconstruction claims for nonmetastatic IBC. Competing risk and Cox regression were used to assess whether IBR was associated with higher breast cancer‐specific mortality (BCSM) or overall mortality (OM). Among 552 936 patients, 1472 (median age 74 years) were diagnosed with IBC and had a mastectomy. Forty‐four patients (3%) underwent IBR. Younger age, a lower Charlson comorbidity score, and a greater median income were predictors of IBR use. Tumor grade, hormone receptor status, and lymph node status were independent predictors of adjusted OM and BCSM. There was no difference by IBR status in BCSM or covariate‐adjusted BCSM (sHR 1.04; CI 0.71‐1.54; *P* = .83 and sHR 1.13; CI 0.84‐1.93; *P* = .58, respectively). Cumulative incidence of OM was lower among IR patients (*P* = .013), and IR did not influence the cumulative incidence of BCSM (*P* = .91). IBR was not associated with increased overall and BCSM mortality. Although further study of IBR in the IBC setting may be of value, these data suggest that IBC should not be considered an absolute contraindication to IBR.

## 1 INTRODUCTION

Inflammatory breast cancer (IBC) is an uncommon and locally advanced cancer accounting for 0.5%‐2% of all newly diagnosed breast cancers in the United States.^1^, ^2^, ^3^, ^4^ Due to malignant infiltration of dermal lymphatics, IBC is typically aggressive, having a poor prognosis and overall survival.^2^ Survival is improved with multimodality therapy that consists of neoadjuvant chemotherapy and postmastectomy radiation therapy.^5^, ^6^, ^7^, ^8^ Most patients without systemic malignancy undergo modified radical mastectomy resulting in increased locoregional control.^3^, ^8^

Traditionally, IBC was considered a relative contraindication for breast reconstruction due to concerns for margin positivity, high risk of recurrence, poor long‐term survival, and concern about potential delay of treatments from surgical complications.^9^, ^10^ The NCCN Breast Cancer Panel recommends delayed breast reconstruction as an option to women with IBC who have undergone a modified radical mastectomy.^9^ However, the surgical paradigm has not been re‐examined as IBC survival outcomes have improved with multimodality treatment. If IBC patients can safely undergo breast reconstruction, they may benefit because of its ability to enhance body image, self‐esteem, and quality of life.^11^, ^12^

Reduction in delays to surgical treatment of breast cancer has the potential to improve overall and disease‐specific survival, comparable to the addition of some standard therapy.^13^ A recent review determined that immediate breast reconstruction (IBR) did not delay initiation of adjuvant chemotherapy.^14^, ^15^ Small, single‐institution studies have suggested that performing delayed or immediate autologous reconstruction in the setting of IBC is safe and does not impact disease‐free and overall survival,^16^, ^17^ but because of its low incidence and poor survival, there is a paucity of literature evaluating the relationship of IBR to survival.

While prior reports have noted that chronologic age^18^, ^19^ and comorbidities^20^ thought to preclude breast reconstruction were some of the reasons for the infrequent rate of mastectomy with IBR, recent studies have demonstrated an increase in the use of postmastectomy IBR for breast cancer patients.^21^, ^22^ Moreover, a recent analysis of the National Cancer Database found that mastectomy and IBR rates are increasing among all patients age 65 years and older. The life expectancy of a women aged 65 years is age 86 years.^23^ Anecdotally we have had patients increasingly request IBR in the IBC setting. This study evaluated survival after treatment of IBC with and without IBR in the ≥65 year old population using one of the largest United States datasets: the Surveillance, Epidemiology, and End Results (SEER)‐Medicare‐linked database, to inform clinical decision‐making and patient counseling, and re‐evaluate management guidelines.

## 2 METHODS

The Fox Chase Cancer Center institutional review board approved the study, and permission to use the SEER‐Medicare database was obtained from the National Cancer Institute (NCI). All sixteen applicable SEER registries were used to increase the external generalizability of the results.

The SEER cancer diagnosis date is specified by only month and year. Medicare claims were searched from the start of the month of diagnosis and for an additional 13 months. Patients were not excluded based on history of prior or other cancer, or on receipt of radiotherapy or chemotherapy. Patients were included if they had nonmetastatic IBC as indicated by AJCC 6th edition stage T4d (“Inflammatory carcinoma”) for cases diagnosed 2004‐2009; or extent of disease code 70 (“Inflammatory carcinoma, including diffuse (beyond that directly overlying the tumor) dermal lymphatic permeation or infiltration”) for IBC diagnosed 1991‐2003. The dates of diagnoses between 1991 until 2009 were chosen to include sufficient length of follow‐up (ie, 5 years). Additionally, the cohort included females diagnosed ≥65 years of age having complete staging and diagnosis date variables, who had Medicare claims for mastectomy to permit the identification of the breast and reconstruction surgery status and dates. Medicare claims codes for surgery were identified to evaluate the interval between mastectomy and reconstruction procedures (Table S1). Surgery dates and receipt of chemotherapy and radiotherapy were derived from physician claims, supplemented by outpatient and inpatient hospital claims. All submitted Medicare claims were reviewed for relevant procedures and dates. Charlson Comorbidity Index (CCI) was estimated from Medicare claims diagnosis codes using a modified method of Klabunde.^24^

Patient demographic, tumor, and treatment characteristics were considered potential confounders, including age at diagnosis, race (per Medicare), marital status, CCI, United States region, census tract poverty level, census tract median income, year of diagnosis, tumor grade, tumor receptor status, lymph node (LN) status, radiotherapy, and chemotherapy use. The association of each characteristic with immediate reconstruction was evaluated using logistic regression.

Two survival outcomes of IBC patients, breast cancer‐specific mortality (BCSM), and overall mortality (OM) were investigated using SEER vital status and cause of death. Competing risk regression mortality analysis was used to evaluate the association of IBR with BCSM while accounting for competing risk of death from other causes, and standardization of follow‐up dates based on date of treatment. Risk comparisons are reported as sub‐distribution hazard ratios (sub‐HR). Similarly, Cox proportional hazards regression was used to estimate hazard ratios [HR] for the OM outcome. Three levels of covariate adjustment were examined: (1) adjustment for age at diagnosis; (2) further adjustment for statistically significant characteristics (*P* < .05) associated with immediate reconstruction; and (3) adjustment for all covariates in Table 3. Comparative cumulative incidence functions illustrate the sub‐HR difference between IBR and no reconstruction with all other covariates held constant. Statistical significance was set at *P *=* *.05 (two‐sided). Analyses were performed using SAS software, version 9.3 (SAS Institute) and Stata software, release 12 (StataCorp 2011).

## 3 RESULTS

### 3.1 Characteristics of inflammatory breast cancer cohort

Among patients in the SEER‐Medicare breast cancer dataset (1991‐2009), we identified 1472 IBC patients meeting inclusion criteria, and 44 IBC patients having complete claims for surgical intervention and treatment (Table 1). Mean age of diagnosis was 75.5 (range 65‐103) years old. The cohort was primarily Caucasian (83.9%), married (36.9%), and healthy with CCI ≤1 (58.5%). Most were living within a 5%‐10% poverty census tract (30%) and reported $25 000‐$50 000 median income. The number of women diagnosed with IBC increased nearly 1.5‐fold each period until 2001‐2004.

**Table 1:** Distribution of patient and oncologic characteristics of inflammatory breast cancer patients from the SEER‐Medicare 1991‐2009 Database (n = 1472)

Demographic characteristics	n	%	Tumor characteristics	n	%
Age at diagnosis (y)			AJCC stage		
Age 65‐69	383	26.0	Stage IIIB (3rd, 6th)	1304	88.6
Age 70‐74	366	24.9	Stage IIIC (6th)	119	8.1
Age 75‐79	289	19.6	NOS or unknown	49	3.3
Age 80‐84	234	15.9	T‐stage		
Age >85	200	13.6	002‐025 mm	123	8.4
Race (Medicare)			026‐050 mm	225	15.3
White	1235	83.9	051‐270 mm	301	20.4
Black	153	10.4	Diffuse	680	46.2
Other	84	5.7	Unknown	143	9.7
Marital status			Grade		
Not married	321	21.8	Well‐differentiated	44	3.0
Married	543	36.9	Moderately‐differentiated	354	24.0
Widowed	608	41.3	Poorly or undifferentiated	867	58.9
Charlson Comorbidity Index			NOS	207	14.1
0	464	31.5	Histology		
1	397	27.0	Inflammatory breast cancer	752	51.1
2‐3	406	27.6	Ductal cancer	529	35.9
4‐11	205	13.9	Other	191	13.0
Region in U.S.^a^			Receptor status		
Northeast	241	16.4	Any positive receptor	772	52.4
South	256	17.4	Negative receptors (ER‐/PR‐)	457	31.0
Midwest	255	17.3	Unknown	243	16.5
West	720	48.9	Lymph nodes examined		
Urban/Rural^b^			None	298	20.2
Big Metro	750	51.0	1‐11	552	37.5
Metro	480	32.6	>12	545	37.0
Urban	84	5.7	Unknown	77	5.2
Less Urban/Rural	158	10.7	Lymph nodes positive		
			No lymph nodes examined	298	20.2
0%‐5%	397	27.0	0	146	9.9
5%‐10%	442	30.0	1‐3	274	18.6
10%‐20%	397	27.0	4‐9	355	24.1
>20%	236	16.0	>10	337	22.9
Median income within census tract			Unknown	62	4.2
<$25 000	155	10.5	Treatment Characteristics		
$25 000‐$50 000	844	57.3	Radiation therapy (XRT)		
$50 000‐$75 000	348	23.6	XRT	893	60.7
>$75 000	125	8.5	No XRT	579	39.3
Year of diagnosis			Chemotherapy status		
1991‐1995	231	15.7	Yes	957	65.0
1996‐2000	344	23.4	No	515	35.0
2001‐2004	485	32.9	Surgery		
2005‐2009	412	28.0	Mastectomy	1472	100.0

a Region groupings are as follows: Northeast (Connecticut and New Jersey); South (Atlanta, rural Georgia, Kentucky, and Louisiana); Midwest (Detroit and Iowa); West (Hawaii, New Mexico, Seattle, Utah and California).

b Urban/rural setting definitions are: Large metro=counties in Metro areas of >/= 1,000,000 population; Metro=counties in metro areas of 250,000 to 1,000,000 population; Urban=urban population >/= 20,000 adjacent or nonadjacent to a metro area; Less urban/rural or rural population of <20,000.

Most patients were Stage IIIB (88.6%) and had T4d presentation (46.2%). The most common tumor biology was poorly differentiated/undifferentiated grade (58.9%), IBC histology (51.1%), and any positive hormone receptor (HR) (52.4%). Nearly 75% had at least 1 lymph node (LN) examined and had at least 1 positive LN in 65.6% of patients. All IBC patients underwent mastectomy (n = 1472), and the majority received chemotherapy (65%) and radiation therapy (60.7%).

### 3.2 Demographic and tumor characteristics associated with immediate breast reconstruction

Immediate breast reconstruction patients (3%, n = 44) were younger compared to their nonreconstructed counterparts (mean = 72.6 vs 75.6 years, *P* = .008) (Table 2). Immediate breast reconstruction was associated with marriage (*P* = .016), lower CCI (*P* = .029), and greater census tract median income (*P* = .024). Trend analyses demonstrated that fewer IBRs occurred with increasing age at diagnosis (*P* = .0050), CCI score (*P* = .0042), and census tract poverty (*P* = .0099). An inverted‐U trend of IBR was associated with increasing median income (*P* = .0033), where the greatest proportion of IBR was performed among patients reporting a median income of $25 000‐$75 000. Associations between IBR and characteristics of IBC patients are delineated in Table S2. Notably, patients with poor health as denoted by a greater CCI score were significantly less likely to undergo IBR (CCI 1 vs 0, OR = 0.77; CCI 2‐3, OR = 0.32; CCI 4‐11, OR = 0.31; *P* = .041). When adjusted for marital status, CCI, and median income on multivariable analyses, the only independent predictor of undergoing IBR was median income (*P* = .047). Analysis of the ICD‐9 implant‐related complications in our cohort was a 7% (n = 3, *P* < .003) rate of mechanical complication due to breast implant in the IBC group undergoing breast reconstruction (n = 44), compared to the no‐reconstruction groups (0%, n = 1428).

**Table 2:** Association of immediate reconstruction with patient and oncologic characteristics in inflammatory breast cancer patients (n = 1472)

	No immediate reconstruction (n = 1428)		Immediate reconstruction (n = 44)		*P* c
	n	%	n	%	
**Patient characteristics**					
Age at diagnosis, mean (SD)	75.6 (7.6)		72.6 (6.1)		.008^d^
Age group at diagnosis					
Ages 65‐69	366	25.6	17	38.6	.091
Ages 70‐74	352	24.6	14	31.8	
Ages 75‐79	282	19.7	a		
Ages 80‐84	230	16.1	a		
Ages >85	198	13.9	a		
Race					
White	1196	83.8	39	88.6	.769
Black	149	10.4	a		
Other	83	5.8	a		
Marital status					
Married	520	36.4	23	52.3	.016
Not married	309	21.6	a		
Widowed	599	41.9	a		
Charlson Comorbidity Index					
0	443	31.0	21	47.7	.029
1	383	26.8	14	31.8	
2‐3	400	28.0	a		
4‐11	202	14.1	a		
Region in U.S.^b^					
Northeast	233	16.3	a		.188
South	250	17.5	a		
Midwest	252	17.6	a		
West	693	48.5	27	61.4	
Urban/Rural^c^					
Large Metro	724	50.7	26	59.1	.778
Metro	467	32.7	13	29.5	
Urban	82	5.7	a		
Less Urban/Rural	155	10.9	a		

0%‐5%	378	26.5	19	43.2	.064
5%‐10%	431	30.2	11	25.0	
10%‐20%	386	27.0	a		
>20%	233	16.3	a		
Median Income within Census tract					
<$25 000	152	10.6	a		.024
$25 000‐$50 000	825	57.8	19	43.2	
$50 000‐$75 000	335	23.5	13	29.5	
>$75 000	116	8.1	a		
Year of Dx					
1991‐1995	223	15.6	a		.786
1996‐2000	332	23.2	a		
2001‐2004	473	33.1	12	27.3	
2005‐2009	400	28.0	12	27.3	
**Oncologic characteristics**					
AJCC stage					
Stage IIIB (3rd, 6th)	1268	88.8	36	81.8	.171
Stage IIIC (6th)	114	8.0	a		
NOS or unknown	46	3.2	a		
T‐stage					
002‐025 mm	122	8.5	a		.542
026‐050 mm	218	15.3	a		
051‐270 mm	292	20.4	a		
Diffuse	656	45.9	24	54.5	
Unknown	140	9.8	a		
Grade					
Well‐differentiated	42	2.9	a		.734
Moderately‐differentiated	341	23.9	13	29.5	
Poorly or undifferentiated	844	59.1	23	52.3	
NOS	201	14.1	a		
Histology					
Inflammatory breast cancer	731	51.2	21	47.7	.580
Ductal cancer	514	36.0	a		
Other	183	12.8	a		
Receptor status					
Any positive receptor	748	52.4	24	54.5	.872
Negative receptors (ER‐/PR‐)	443	31.0	a		
Unknown	237	16.6	a		
Lymph nodes examined					
None	288	20.2	a		.815
1‐11	536	37.5	16	36.4	
>12	528	37.0	17	38.6	
Unknown	76	5.3	a		
Lymph nodes positive					
No lymph nodes examined	288	20.2	a		.887
0	141	9.9	a		
1‐3	264	18.5	a		
4‐9	347	24.3	a		
>10	327	22.9	a		
Unknown	61	4.3	a		
Radiation therapy (XRT)					
XRT	861	60.3	32	72.7	.096
No XRT	567	39.7	12	27.3	
Chemotherapy status					
Yes	927	64.9	30	68.2	.655
No	501	35.1	14	31.8	

a As per National Cancer Institute Surveillance, Epidemiology, and End Results‐Medicare requirements, cells containing <11 individuals and any cells making them calculable have been censored.

b Region groupings are as follows: Northeast (Connecticut and New Jersey); South (Atlanta, rural Georgia, Kentucky, and Louisiana); Midwest (Detroit and Iowa); West (Hawaii, New Mexico, Seattle, Utah and California).

c *P*‐value for difference in characteristic by reconstruction status using Chi‐square test or Fischer’s exact test.

d *P*‐value for age as a continuous variable using *t*‐test.

### 3.3 Breast cancer‐specific and overall mortality

In univariate analysis (Table 3), IBR was not associated with greater BCSM (sHR = 1.04, CI 0.71‐1.54; *P* = .83). Factors associated with greater BCSM included earlier year of IBC diagnosis (*P* = .0016), histologic grade other than well‐differentiated (*P* < .0001), ER‐negative/PR‐negative or unknown receptor status (sHR = 2.02 and 1.47, respectively, *P* < .0001), increasing number of positive LN (*P* < .0001), and either neoadjuvant or adjuvant chemotherapy (*P* = .0027). Having 1‐11 or >12 LN examined was associated with lower BCSM. Breast cancer‐specific mortality was not associated with age of diagnosis, race, marital status, U.S. region, socioeconomic factors, CCI, and radiation therapy.

**Table 3:** Univariable and multivariable models of breast cancer‐specific mortality (BCSM) in inflammatory breast cancer patients undergoing immediate reconstruction (n = 1472)

	Total (n = 1472)	BCSM (n = 710)	UVA			MVA		
			sHR	95% CI	*P*‐value	sHR	95% CI	*P*‐value
Immediate reconstruction								
Yes	44	23	1.04	[0.71, 1.54]	**.8295**	1.14	[0.71, 1.76]	**.5472**
No	1428	687	1.00	—		1.00	—	
***Patient characteristics***								
Age at diagnosis			1.10	[0.99, 1.01]	.8406	1.01	[1.00, 1.02]	**.0992**
Race (Medicare)					**.9337**			
White	1235	597	1.00	—				
Black	153	76	1.04	[0.81, 1.33]	.737			
Other	84	37	0.98	[0.70, 1.37]	.8965			
Marital status					**.5991**			
Not married	273	140	1.00	—				
Married	543	251	0.87	[0.71, 1.06]	.1718			
Widowed	608	296	0.91	[0.75, 1.11]	.3482			
Unknown	48	23	0.92	[0.60, 1.41]	.7077			
Charlson Comorbidity Index					**.7118**			
0	464	237	1.00	—				
1	397	186	0.99	[0.82, 1.19]	.9123			
2‐3	406	189	1.00	[0.83, 1.20]	.9714			
4‐11	205	98	1.14	[0.89, 1.45]	.2921			
Region of U.S.^b^					**.1601**			
Northeast	241	123	1.00	—				
South	256	119	0.93	[0.73, 1.20]	.5797			
Midwest	255	142	1.01	[0.80, 1.28]	.9122			
West	720	326	0.84	[0.68, 1.03]	.0921			
Census Tract Poverty					**.8439**			
0%‐5%	397	187	1.00	—				
5%‐10%	442	213	1.07	[0.88, 1.30]	.5113			
10%‐20%	397	194	1.08	[0.89, 1.31]	.4468			
>20%	236	116	1.09	[0.87, 1.38]	.4523			
Median Income					**.563**			
<$25 000	155	84	1.13	[0.89, 1.42]	.3218			
$25 000‐$50 000	844	406	1.00	—				
$50 000‐$75 000	348	165	1.02	[0.85, 1.22]	.8276			
>$75 000	125	55	0.88	[0.67, 1.17]	.3849			
Year of diagnosis					**.0016**			**.0003**
1991‐1994	172	110	1.00	—		1.00	—	
1995‐1997	177	15	0.93	[0.72, 1.21]	.6037	0.80	[0.61, 1.05]	
1998‐2000	226	137	0.93	[0.73, 1.17]	.5251	0.77	[0.60, 0.99]	
2001‐2003	374	186	0.75	[0.60, 0.94]	.0142	0.68	[0.53, 0.86]	
2004‐2006	337	130	0.67	[0.52, 0.85]	.0013	0.57	[0.44, 0.74]	
2007‐2009	186	42	0.61	[0.43, 0.87]	.0065	0.53	[0.37, 0.75]	
***Oncologic characteristics***								
Grade					**<.0001**			**.0005**
Well‐differentiated	44	a	1.00	—		1.00	—	
Moderately‐differentiated	354	144	2.45	[1.30, 4.60]	.0055	2.72	[1.45, 5.13]	
Poorly or undifferentiated	867	464	3.68	[1.98, 6.83]	<.0001	3.19	[1.71, 5.95]	
NOS	207	a	2.73	[1.44, 5.19]	.0021	2.51	[1.32, 4.79]	
Receptor status					**<.0001**			**<.0001**
Any positive receptor	772	310	1.00	—		1.00	—	
Negative receptors (ER‐/PR‐)	457	269	2.02	[1.71, 2.38]	<.0001	2.01	[1.69, 2.39]	
Unknown	243	131	1.47	[1.20, 1.80]	.0002	1.37	[1.11, 1.69]	
Lymph nodes examined					**.0022**			**<.0001**
None	298	165	1.00	—		1.00	—	
1‐11	552	251	0.78	[0.64, 0.95]	.0128	0.43	[0.30, 0.63]	
>12	545	247	0.79	[0.65, 0.96]	.018	0.32	[0.21, 0.47]	
Unknown	77	47	1.27	[0.91, 1.77]	.1605	0.47	[0.22, 1.03]	
Lymph nodes positive					**<.0001**			**<.0001**
No lymph nodes examined	146	36	1.00	—		1.00	—	
0	274	97	1.43	[0.98, 2.09]	.0675	1.64	[1.11, 2.42]	
1‐3	355	172	3.01	[2.11, 4.29]	<.0001	4.05	[2.76, 5.94]	
4‐9	337	198	2.22	[1.55, 3.18]	<.0001	2.54	[1.76, 3.66]	
>10	62	42	3.82	[2.42, 6.04]	<.0001	3.51	[1.55, 7.97]	
***Treatment characteristics***								
Radiation therapy (XRT)								
XRT	893	443	1.08	[0.93, 1.26]	**.3003**			
No XRT	579	267	1.00	—				
Pre‐Op XRT								
Yes	63	34	1.09	[0.77, 1.54]	**.649**			
No	1409	676	1.00	—				
Post‐Op XRT								
Yes	839	414	1.07	[0.92, 1.24]	**.3722**			
No	633	296	1.00	—				
Chemotherapy status								
Yes	957	489	1.28	[1.09, 1.50]	**.0027**			
No	515	221	1	—				
Pre‐Op chemotherapy								
Yes	696	362	1.24	[1.07, 1.43]	**.0040**	1.36	[1.14, 1.61]	.0006
No	776	348	1.00	—		1.00	—	
Post‐Op Chemotherapy								
Yes	723	373	1.19	[1.03, 1.38]	**.0206**	1.04	[0.8, 1.23]	.638
No	749	337	1.00	—		1.00	—	

*P*‐value in bold denote Wald joint Chi‐square tests for overall significance of the predictor in the model.

*P*‐value not in bold are for pairwise comparisons with the referent.

a As per National Cancer Institute Surveillance, Epidemiology, and End Results‐Medicare requirements, cells containing <11 individuals and any cells making them calculable have been censored.

b Region groupings are as follows: Northeast (Connecticut and New Jersey); South (Atlanta, rural Georgia, Kentucky, and Louisiana); Midwest (Detroit and Iowa); West (Hawaii, New Mexico, Seattle, Utah and California).

In multivariate analysis (Table 3) including IBR and significant univariate factors, IBR was not associated with BCSM (sHR = 1.14, CI 0.71‐1.76; *P* = .55). Independent BCSM predictors were year of IBC diagnosis (*P* = .0003), histologic grade (*P* = .0005), HR status (*P* < .0001), number of LN examined (*P* < .0001), number of positive LN (*P* < .0001), and neoadjuvant chemotherapy (*P* = .0006). Aggressive histologic grade and HR status were associated with increased risk of BCSM. Compared to well‐differentiated histologic grade, there is a threefold BCSM risk with poorly differentiated or undifferentiated grade IBC (sHR 3.19, CI 1.71‐5.95, *P* = .005), and a twofold increase of BCSM risk with ER‐negative/PR‐negative hormone status (sHR 2.01, CI 1.69‐2.39, *P* = .0005).

In univariate analysis (Table 4), IBR was associated with lower OM (HR = 0.63, CI 0.42‐0.92; *P* = .018). Factors associated with increased OM included older age at diagnosis (*P* < .0001), single or widowed status (*P* = .0005), higher CCI (*P* < .0001), poorer histologic grade (*P* = .0008), negative or unknown HR status (*P* < .0001), unknown or no LN examined (*P* < .0001), increased number of positive LN (*P* < .0001), no neoadjuvant nor adjuvant radiation therapy (*P* < .0001), no neoadjuvant chemotherapy (*P* < .0010), and no adjuvant chemotherapy (*P* < .0001). Overall mortality did not differ by race, U.S. region, census tract poverty, median income, and year of IBC diagnosis.

**Table 4:** Univariable and multivariable models of overall mortality (OM) in inflammatory breast cancer patients undergoing immediate reconstruction (n = 1472)

	n (n = 1472)	OM (n = 1056)	UVA			MVA		
			HR	95% CI	*P*‐value	HR	95% CI	*P*‐value
Immediate reconstruction								
Yes	44	26	0.63	[0.42, 0.92]	.018	0.82	[0.55, 1.21]	**.319**
No	1428	1030	1.00	—				
**Patient characteristics**								
Age at diagnosis			1.04	[1.03, 1.05]	**<.001**	1.03	[1.02, 1.04]	**<.001**
Race (Medicare)					**.368**			
White	1235	885	1.00	—				
Black	153	117	1.11	[0.92, 1.35]	.285			
Other	84	54	0.89	[0.68, 1.17]	.404			
Marital status					**<.001**			**.861**
Not married	273	192	1.00	—		—		
Married	543	359	0.84	[0.70, 1.00]	0.051	1.11	[0.92, 0.77]	
Widowed	608	472	1.13	[0.95, 1.33]	0.164	1.14	[0.96, 0.81]	
Unknown	48	33	0.94	[0.65, 1.36]	0.754	1.39	[0.96, 0.66]	
Charlson Comorbidity Index					**<.001**			**<.001**
0	464	314	1.00	—			1.00	—
1	397	265	1.20	[1.02, 1.42]	.028	1.15	[1.00, 1.39]	
2‐3	406	315	1.68	[1.44, 1.97]	<.001	1.50	[1.29, 1.79]	
4‐11	205	162	2.25	[1.86, 2.73]	<.001	1.98	[1.64, 2.43]	
Region of U.S.^a^					**.057**			
Northeast	241	183	1.00	—				
South	256	166	0.85	[0.69, 1.05]	.135			
Midwest	255	197	0.79	[0.64, 0.97]	.021			
West	720	510	0.80	[0.68, 0.95]	.010			
Census Tract Poverty					**.366**			
0%‐5%	397	284	1.00	—				
5%‐10%	442	312	1.04	[0.87, 1.22]				
10%‐20%	397	279	1.03	[0.87, 1.22]				
>20%	236	181	1.18	[0.98, 1.42]				
Median Income					**.287**			
<$25 000	155	131	1.15	[0.95, 1.39]	.160			
$25 000‐$50 000	844	602	1.00	—				
$50 000‐$75 000	348	242	1.01	[0.87, 1.18]	.857			
>$75 000	125	81	0.88	[0.69, 1.10]	.261			
Year of diagnosis					**.396**			
1991‐1994	172	165	1.00	—				
1995‐1997	177	155	0.91	[0.73, 1.14]	.412			
1998‐2000	226	200	0.99	[0.80, 1.22]	.907			
2001‐2003	374	284	0.87	[0.71, 1.06]	.161			
2004‐2006	337	196	0.88	[0.71, 1.09]	.233			
2007‐2009	186	56	0.76	[0.56, 1.04]	.085			
***Oncologic characteristics***								
Grade					**<.001**			**.032**
Well‐differentiated	44	28	1.00	—		1.00	—	
Moderately‐differentiated	354	238	1.23	[0.83, 1.82]	.298	1.29	[0.87, 1.83]	
Poorly or undifferentiated	867	644	1.57	[1.08, 2.29]	.020	1.48	[1.01, 2.11]	
NOS	207	146	1.26	[0.84, 1.89]	.260	1.22	[0.81, 1.75]	
Receptor status					**<.001**			**<.001**
Any positive receptor	772	520	1.00	—		1.00	—	
Negative receptors (ER‐/PR‐)	457	334	1.52	[1.33, 1.75]	<0.001	1.70	[1.47, 1.95]	
Unknown	243	202	1.40	[1.19, 1.64]	<0.001	1.37	[1.16, 1.59]	
Lymph nodes examined					**<.001**			**<.001**
None	298	241	1.21	[1.03, 1.42]	0.020	1.00	—	
1‐11	552	384	1.00	—		0.49	[0.38, 0.65]	
>12	545	366	0.93	[0.81‐1.08]	0.347	0.36	[0.27, 0.48]	
Unknown	77	65	1.61	[1.24‐2.10]	<0.001	0.39	[0.21, 0.73]	
Lymph nodes positive					**<.001**			**<.001**
No lymph nodes examined	146	70	1.00	—		1.00	—	
0	274	169	1.38	[1.04, 1.83]		1.49	[1.12, 1.98]	
1‐3	355	252	1.93	[1.48, 2.51]		3.31	[2.49, 4.41]	
4‐9	337	268	2.60	[2.00, 3.39]		2.10	[1.60, 2.75]	
>10	62	56	3.34	[2.34, 4.75]		4.23	[2.22, 8.09]	
**Treatment characteristics**								
Radiation therapy (XRT)								
XRT	893	604	0.74	[0.65, 0.84]	**<.001**			
No XRT	579	452	1.00	—				
Pre‐Op XRT								
Yes	63	53	1.09	[0.82, 1.43]	.559			
No	1409	1003	1.00	—				
Post‐Op XRT								
Yes	839	559	0.74	[0.66, 0.84]	**<.001**	0.83	[0.72, 0.95]	.007
No	633	497	1.00	—		1.00	—	
Chemotherapy status								
Yes	957	643	0.73	[0.65, 0.83]	**<.001**			
No	515	413	1.00	—				
Pre‐Op Chemotherapy								
Yes	696	462	0.81	[0.72, 0.92]	**.001**	1.10	[0.95, 1.27]	.220
No	776	594	1.00	—		1.00	—	
Post‐Op Chemotherapy								
Yes	723	485	0.77	[0.68, 0.87]	**<.001**	0.86	[0.75, 0.99]	.034
No	749	571	1.00	—		1.00	—	

*P*‐value in bold denote Wald joint Chi‐square tests for overall significance of the predictor in the model.

*P*‐value not in bold are for pairwise comparisons with the referent.

a Region groupings are as follows: Northeast (Connecticut and New Jersey); South (Atlanta, rural Georgia, Kentucky, and Louisiana); Midwest (Detroit and Iowa); West (Hawaii, New Mexico, Seattle, Utah and California).

With adjustment for covariates, IBR was no longer associated with lower OM (HR = 0.82, CI 0.55‐1.21; *P* = .319) (Table 4). Independent predictors of OM were: age of diagnosis (*P* < .0001), CCI (*P* < .0001), histologic grade (*P* = .0318), HR status (*P* < .0001), number of LN examined (*P* < .0001), number of positive LN (*P* < .0001), adjuvant radiation therapy (*P* = .0066), and adjuvant chemotherapy (*P* = .0343). Similar to BCSM, observations of aggressive histologic grade and HR status were associated with increased risk of OM. Similar relationships were demonstrated between BCSM and both examined and positive LN.

To examine the association of chemotherapy and radiation therapy with BCSM over time, we stratified the cohort into 4 time periods, and looked at differences in BCSM by treatment within each period. The cumulative incidence of BCSM was significantly greater in chemotherapy patients in the first period (1991‐1995, Gray’s test *P* = .0008). Each successive period (1996‐2000; 2001‐2004; 2005‐2009) did not demonstrate a difference by chemotherapy or radiation therapy status (each Gray’s test *P* > .05). When examined in the multivariable modeling, the interaction between time period and additional treatment was not statistically significant.

### 3.4 Cumulative incidence of breast cancer‐specific and overall mortality associated with immediate breast reconstruction

**Figure 1:**
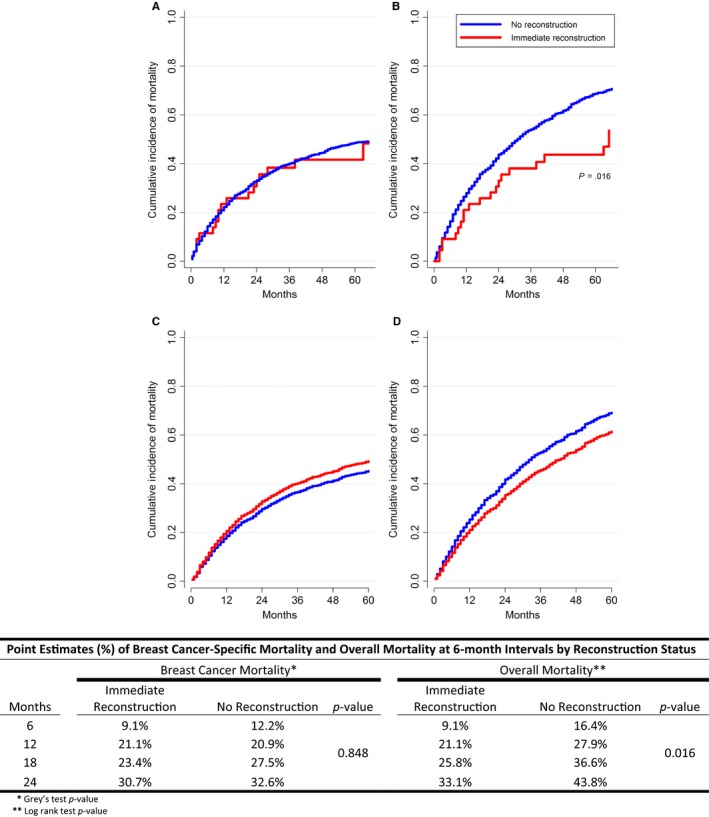
A‐D, Cumulative incidence of unadjusted and adjusted breast cancer‐specific mortality (BCSM) and overall mortality (OM) associated with immediate reconstruction status accounting for competing risk of other causes of death (A, Unadjusted BCSM; B, Unadjusted OM; C, Adjusted BCSM; D, Adjusted OM)

There was no difference in BCSM or adjusted BCSM (sHR 1.04; CI 0.71‐1.54; *P* = .83 and sHR 1.13; CI 0.84‐1.93; *P* = .582, respectively) when comparing reconstruction status (Figure 1A,C). Immediate breast reconstruction was associated with a lower OM risk compared to patients not having IBR (hazard ratio [HR] 0.63; CI 0.42‐0.92; *P* = .018), but this difference was no longer significant after adjusting for age, comorbidities, and other covariates (Figure 1B,D). Reconstruction status did not affect BCSM or OM regardless of HR status or histologic grade (data not shown).

## 4 DISCUSSION

Historically, the 5‐year overall survival for IBC following mastectomy alone was 2%‐4%.^25^ Strategies for IBC treatment transitioned to radiation therapy alone or combined with surgery, but did not confer survival improvement. Recently, advances in multimodal therapy have improved the 5‐year overall survival rate to 34%‐47%.^6^ Multimodal therapy consists of neoadjuvant chemotherapy, modified radical mastectomy, and postmastectomy radiotherapy.^6^, ^7^ Given the prognostic improvement, IBC that was once a relative contraindication to reconstruction has been given new reconsideration.

A few single‐institution studies have investigated whether breast reconstruction affects oncologic and survival outcomes among IBC patients.^3^, ^16^, ^17^ Chin et al^3^ reported no difference in disease‐free or overall survival when comparing 22 IBC patients to nonreconstructed historical controls. The median disease‐free and overall survival rates were 19 and 22 months, respectively. No differences in oncologic outcomes were observed between 14 IBR patients and the remaining delayed reconstruction patients. Chang et al^16^ performed a 12‐year retrospective review of 830 IBC patients and demonstrated improved overall survival and similar complication rates when comparing 59 free autologous reconstruction IBC patients to their nonreconstructed counterparts. Of note, only 7 patients underwent IBR with free tissue. When examining a large, multicenter cohort of postmastectomy patients undergoing autologous breast reconstruction, Song and colleague demonstrated that women over age 65 years, accounting for 3% of the entire cohort, tolerated reconstruction with high satisfaction, and had no significant differences in postoperative complications as compared to younger patients.^26^ Moreover, whether breast reconstruction was implant‐based or autologous tissue, advanced age did worsen recovery or long‐term morbidity.^27^ Recently, Simpson et al^17^ identified 16 IBC women who received IBR, where the majority were tissue expander‐based procedures. Similar to their 6% implant‐related complication rate,^17^ we report a 7% complication rate in our cohort of IBC patients. While reconstruction‐associated complications did occur, IBR was not associated with elevated recurrence or lowered survival. These studies suggest that both delayed and immediate breast reconstruction are oncologically safe, well tolerated, and do not impact survival.

To our knowledge, the only 2 studies that evaluate survival in any meaningful manner, which are good studies, but do markedly differ from ours. The first paper by Chang and colleagues^16^ is a single‐institution retrospective analysis of 59 IBC patients who primarily underwent delayed autologous reconstruction. In this study, there was no control group comparison for mortality. The second study by Simpson and colleagues^17^ is a single‐institution review of breast reconstruction in IBC patients with a control group that demonstrated that immediate breast reconstruction was not associated with decreased survival. However, this study’s conclusion was based on the limited IBC cohort of 16 patients who underwent immediate reconstruction.

We performed a population‐based study using the SEER‐Medicare dataset to evaluate mortality in a nationally representative cohort of IBC patients, and beyond recent studies, we confirmed an association between IBR status and IBC outcomes.^28^, ^29^, ^30^ Agarwal et al demonstrated in their SEER database study of all breast cancer postmastectomy patients that reconstructed women had a lower hazard of OM when compared to nonreconstructed women.^29^ In a follow‐up study, they demonstrated greater breast cancer‐specific survival in patients that underwent reconstruction, although this may have been due to selection bias.^30^ These studies validate our unadjusted finding that OM was improved follow mastectomy and IBR. Although we also demonstrated that IBR did not confer an OM benefit after adjusting for potential confounders in the 44 postmastectomy IBC patients when compared to the 1428 nonreconstructed IBC women, we found that IBC patients who have IBR after mastectomy have similar BCSM in comparison with IBC patients who do not undergo reconstruction. No difference in mortality was present after adjustment. To our knowledge, this represents the largest study of mortality in IBR IBC patients.

Our evaluation demonstrated that more recent IBC diagnosis was associated with a progressive reduction in hazard of death. Improvement in BCSM is multifactorial. Over recent decades, targeted and less toxic chemotherapeutic protocols have been developed.^31^ The most recent 2016 NCCN guideline for IBC recommends preoperative systemic chemotherapy using anthracycline‐ and taxane‐based therapy with the addition of HER2‐targeted therapy in tumors with HER‐2/neu overexpression.^9^ Additional improvements appear to be due to advances in diagnostic technology that detect IBC earlier, which translate to earlier treatment, and adoption of multimodality treatment to improve oncologic outcomes for IBC patients.^5^, ^6^, ^7^, ^8^

While our review of the SEER‐Medicare dataset was not designed to evaluate the IBR itself or most optimal method of reconstruction in IBC patients, we did find that performance of IBR overall does not present the oncologic concerns previously theorized. Additionally, this analysis confirmed prior findings^29^, ^30^ that tumor characteristics, such as histological grade, positive receptor status, and lymph node involvement, are independent predictors of adjusted OM and BCSM in IBC patients undergoing IBR. Women with IBC were more likely to undergo mastectomy and IBR if they were younger, married, healthier, and had greater median income. Yet, the single predictor of undergoing IBR was median income. This may suggest that wealthier areas have improved access to care and have the opportunity to benefit from IBR to enhance body image, self‐esteem, and quality of life without compromising oncologic and survival outcomes or increasing complications.^11^, ^12^, ^16^, ^17^

The unique nature of this study is its national representation of patient demographics and tumor characteristics allowing greater generalizability of our results nationally via the Medicare population. While this cohort is 65 and over, there is little reason why our results should be inapplicable to a younger population, but as recurrence rates can be slightly higher in a younger cohort, additional study in this group is indicated. Epidemiologic studies report that IBC accounts for 0.5%‐2% of invasive breast cancers and is diagnosed at an earlier age (mean of 59 years vs 62 years).^2^, ^4^ We identified approximately 0.3% (n = 1472) of nonmetastatic IBC patients among the overall breast cancer SEER‐Medicare cohort (n = 552 936). Our IBC study cohort had a greater mean age at diagnosis of 75.5 years compared to the overall SEER invasive breast cancer cohort (mean age 59 years).^4^ In comparison with the overall age of invasive breast cancer SEER patients, we observed a smaller, older cohort of IBC patients from examining only the SEER‐Medicaid linked database. While our conclusions are limited to an older IBC population, we anticipate that an inclusive cohort of IBC patients will demonstrate a survival benefit with IBR.

Elderly postmastectomy breast reconstruction patients are underrepresented,^27^ and even more so in the subgroup of IBC patients. This is evident in our small sample size, and this is likely due to the fact that IBR is presently considered contraindicated for IBC patients. But as the United States population overall and IBC patients specifically are showing an increasing longevity, denial of IBR in the IBC setting for historic reasons may no longer be justified and should remain an option. We believe our study is a good starting point for further consideration and analysis, and our data are the best evidence to date that IBR has little impact on mortality.

Although the present study was not designed to analyze the perioperative complications associated with and without IBR in the elderly cohort, reconstruction remains safe and is performed frequently in those over 65. While postmastectomy radiation is known to increase the risk of postmastectomy IBR complications,^32^ our analysis of SEER‐Medicare data results suggest that age, Charlson comorbidity scores, and radiation therapy do not increase risk of BCSM. We found that fewer older IBC patients in the SEER‐Medicare dataset (Table 1) received radiation therapy than expected (40%). To determine whether this was unique to the older IBC cohort, we re‐analyzed the SEER variable for radiation use (as vs Medicare claims codes) for the overall breast cancer population. We found that indeed a large proportion did not receive radiation (47%), and this finding was similar to our results. Similar findings were demonstrated after review of lymph node examination. Although these numbers seem low, the older IBC population is a group in whom selective omission of various treatments may occur more frequently. Our analysis attempts to compensate for such findings, by adjusting for adjuvant therapy usage or omission, so these numbers should have minimal impact on our analysis. Moreover, we believe that such numbers, if they had an effect, would skew the data away from our conclusion of similarity between groups. Yet we still found no difference, further strengthening our argument about safety. Therefore, our data offer guidance to counseling older IBC patients regarding the impact of surgical treatment on postoperative outcomes and BCSM.

Although this dataset does not include every possible comorbidity, we utilized Charlson Comorbidity Index to adjust for health issues that may affect mortality. Moreover, while our focus is on the issue of survival and outcomes with or without reconstruction, we recognize that components of treatment may impact outcomes related to reconstruction (eg, radiation‐related complications of implant breast reconstruction). Now that we have demonstrated that cancer outcomes are not compromised by the reconstruction, the secondary consideration of how to avoid or minimize such complications in the setting of radiotherapy should be evaluated in additional studies.

Finally, while we can see no reason why these results would markedly differ from a similar study in a younger cohort, confirmation in those under 65 would be desirable. Although younger patients have a higher recurrence risk, the added risk is present whether patients have mastectomy or breast conservation. We therefore do not think that the performance of breast reconstruction in younger women should change outcomes or show results any differently than it does here. Such a marked treatment difference by age would be a highly unique finding. Despite these limitations, our current analysis of a national cohort of IBC patients represents an important step in examining the interaction of IBR and BCSM.

As women with IBC now undergo multimodal treatment that confers a disease‐specific mortality that is markedly improved compared with outcomes that occurred when IBR was first considered contraindicated, these results should provide reassurance to patients and multidisciplinary teams that the addition of IBR does not have a negative impact on BCSM. Ultimately just because we can do something does not mean we should*,* but our evaluation suggests that IBR is safe, and should not be considered a contraindication in carefully selected IBC patients.

## CONFLICT OF INTEREST

There are no conflicts of interest.
