# Supplementary material for: Immediate breast reconstruction for women having inflammatory breast cancer in the United States
Source: Cancer Med. 2018 May 15;7(7):2887–902. doi: 10.1002/cam4.1546 (PMC6051180; doi:10.1002/cam4.1546)
Supplement: Supplementary file 1 [file CAM4-7-2887-s001.docx]

**Supplementary Table 1. CPT and ICD-9 Codes for Surgery and Reconstruction Procedures**

| **Reconstruction Codes** |  |
| --- | --- |
| **CPT codes** |  |
|  | 19324, 19325, 19340, 19342, 19357, 19361, 19362, 19364, 19366, 19367, 19368, 19369, 19370, 19371, 19380, 19396 |
| **ICD-9 Codes** |  |
|  | 85.33, 85.35, 85.5, 85.50, 85.53, 85.54, 85.7, 85.70, 85.71, 85.72, 85.73, 85.74, 85.75, 85.76, 85.79, 84.84, 85.85, 85.93, 85.94, 85.95, 84.95 |
| **Mastectomy Codes** |  |
| **CPT Codes** |  |
|  | 19180, 19182, 19200, 19220, 19240, 19303, 19304, 19305, 19306, 19307 |
| **ICD-9 Codes** |  |
|  | 85.34, 85.36, 85.40, 85.41, 85.42, 85.43, 85.44, 85.45, 85.46, 85.47, 85.48 |

Abbreviations: CPT (current procedural terminology) and ICD-9 (International Classification of Diseases, Ninth Revision)

**Supplementary Table 2** Univariate and Multivariate Analyses of Association of Immediate Reconstruction and Characteristics of Inflammatory Breast Cancer Patients (n=1,472)

|  |  | **UVA** | | | |  | **MVA** | | | |
| --- | --- | --- | --- | --- | --- | --- | --- | --- | --- | --- |
|  | n | OR | 95% CI | | *p*-value |  | OR | 95% CI | | *p*-value |
| **Age at Diagnosis, continuous** | | 0.73 | [0.58, 0.92] | | 0.009 |  | 0.83 | [0.65, 1.06] | | 0.139 |
| OR for 5-year difference | |  |  |  |  |  |  |  |  |  |
| **Race** | |  |  |  | 0.590 |  |  |  |  |  |
| White | 1,235 | 1.00 | - | |  |  |  |  |  |  |
| Black | 153 | 0.82 | [0.29, 2.34] | |  |  |  |  |  |  |
| Other | 84 | 0.37 | [0.05, 2.72] | |  |  |  |  |  |  |
| **Marital Status** | |  |  |  | 0.022 |  |  |  |  | 0.141 |
| Not married | 321 | 2.58 | [1.08, 6.20] | |  |  | 2.14 | [0.87, 5.30] | |  |
| Married | 543 | 2.94 | [1.35, 6.42] | |  |  | 2.20 | [0.97, 4.99] | |  |
| Widowed | 608 | 1.00 | - | |  |  | 1.00 | - | |  |
| **Charlson Comorbidity Index** | |  |  |  | 0.041 |  |  |  |  | 0.141 |
| 0 | 464 | 1.00 | - | |  |  | 1.00 | - | |  |
| 1 | 397 | 0.77 | [0.39, 1.54] | |  |  | 0.87 | [0.43, 1.76] | |  |
| 2-3 | 406 | 0.32 | [0.13, 0.79] | |  |  | 0.40 | [0.16, 1.01] | |  |
| 4-11 | 205 | 0.31 | [0.09, 1.06] | |  |  | 0.38 | [0.11, 1.29] | |  |
| **Region in U.S.*^b^*** |  |  |  |  | 0.221 |  |  |  |  |  |
| Northeast | 241 | 0.88 | [0.39, 1.97] | |  |  |  |  |  |  |
| South | 256 | 0.62 | [0.25, 1.51] | |  |  |  |  |  |  |
| Midwest | 255 | 0.31 | [0.09, 1.02] | |  |  |  |  |  |  |
| West | 720 | 1.00 | - | |  |  |  |  |  |  |
| **Urban / Rural*^c^*** |  |  |  |  | 0.694 |  |  |  |  |  |
| Big Metro | 750 | 1.00 | - | |  |  |  |  |  |  |
| Metro | 480 | 0.78 | [0.39, 1.52] | |  |  |  |  |  |  |
| Urban | 84 | 0.68 | [0.16, 2.91] | |  |  |  |  |  |  |
| Less Urban / Rural | 158 | 0.54 | [0.16, 1.80] | |  |  |  |  |  |  |
| **Poverty, % within Census tract** | |  |  |  | 0.080 |  |  |  |  |  |
| 0-5% | 397 | 1.00 | - | |  |  |  |  |  |  |
| 5-10% | 442 | 0.51 | [0.24, 1.08] | |  |  |  |  |  |  |
| 10-20% | 397 | 0.57 | [0.27, 1.21] | |  |  |  |  |  |  |
| >20% | 236 | 0.26 | [0.08, 0.88] | |  |  |  |  |  |  |
| **Median Income within Census tract** | |  |  |  | 0.022 |  |  |  |  | 0.047 |
| <$25,000 | 155 | 1.00 | - | |  |  | 1.00 | - | |  |
| $25,000-$50,000 | 844 | 1.17 | [0.34, 3.99] | |  |  | 1.03 | [0.30, 3.58] | |  |
| $50,000-$75,000 | 348 | 1.97 | [0.55, 7.00] | |  |  | 1.66 | [0.46, 6.04] | |  |
| >$75,000 | 125 | 3.93 | [1.04, 14.85] | |  |  | 3.24 | [0.84, 12.44] | |  |
| **Year of Diagnosis** |  |  |  |  | 0.821 |  |  |  |  |  |
| 1991-1995 | 231 | 1.00 | - | |  |  |  |  |  |  |
| 1996-2000 | 344 | 1.01 | [0.41, 2.50] | |  |  |  |  |  |  |
| 2001-2004 | 485 | 0.71 | [0.29, 1.75] | |  |  |  |  |  |  |
| 2005-2009 | 412 | 0.84 | [0.34, 2.08] | |  |  |  |  |  |  |
| **AJCC stage** |  |  |  |  | 0.301 |  |  |  |  |  |
| Stage IIIB (3rd,6th) | 1304 | 1.00 | - | |  |  |  |  |  |  |
| Stage IIIC (6th) | 119 | 0.43 | [0.13, 1.46] | |  |  |  |  |  |  |
| NOS or unknown | 49 | 0.67 | [0.15, 2.93] | |  |  |  |  |  |  |
| **T-stage** |  |  |  |  | 0.603 |  |  |  |  |  |
| 002-025 mm | 123 | 0.22 | [0.03, 1.67] | |  |  |  |  |  |  |
| 026-050 mm | 225 | 0.88 | [0.37, 2.07] | |  |  |  |  |  |  |
| 051-270 mm | 301 | 0.84 | [0.39, 1.83] | |  |  |  |  |  |  |
| Diffuse | 680 | 1.00 | - | |  |  |  |  |  |  |
| Unknown | 143 | 0.59 | [0.17, 1.97] | |  |  |  |  |  |  |
| **Grade** |  |  |  |  | 0.737 |  |  |  |  |  |
| Well-differentiated | 44 | 1.75 | [0.40, 7.66] | |  |  |  |  |  |  |
| Moderately-differentiated | 354 | 1.40 | [0.70, 2.79] | |  |  |  |  |  |  |
| Poorly- or un-differentiated | 867 | 1.00 | - | |  |  |  |  |  |  |
| NOS | 207 | 1.10 | [0.44, 2.73] | |  |  |  |  |  |  |
| **Histology** |  |  |  |  | 0.584 |  |  |  |  |  |
| Inflammatory breast cancer | 752 | 1.00 | - | |  |  |  |  |  |  |
| Ductal cancer | 529 | 1.02 | [0.52, 1.99] | |  |  |  |  |  |  |
| Other | 191 | 1.52 | [0.66, 3.49] | |  |  |  |  |  |  |
| **Receptor Status** | |  |  |  | 0.873 |  |  |  |  |  |
| Any positive receptor | 772 | 1.00 | - | |  |  |  |  |  |  |
| Negative receptors (ER-/PR-) | 457 | 0.98 | [0.50, 1.92] | |  |  |  |  |  |  |
| Unknown | 243 | 0.79 | [0.32, 1.95] | |  |  |  |  |  |  |
| **Lymph nodes examined** | |  |  |  | 0.827 |  |  |  |  |  |
| None | 298 | 0.44 | [0.06, 3.37] | |  |  |  |  |  |  |
| 1-11 | 552 | 1.00 | - | |  |  |  |  |  |  |
| >12 | 545 | 1.16 | [0.52, 2.60] | |  |  |  |  |  |  |
| Unknown | 77 | 1.08 | [0.54, 2.16] | |  |  |  |  |  |  |
| **Lymph nodes positive** | |  |  |  | 0.892 |  |  |  |  |  |
| No lymph nodes examined | 298 | 0.98 | [0.33, 2.92] | |  |  |  |  |  |  |
| 0 | 146 | 1.00 | - | |  |  |  |  |  |  |
| 1-3 | 274 | 1.07 | [0.36, 3.19] | |  |  |  |  |  |  |
| 4-9 | 355 | 0.65 | [0.21, 2.02] | |  |  |  |  |  |  |
| >10 | 337 | 0.86 | [0.29, 2.57] | |  |  |  |  |  |  |
| Unknown | 62 | 0.46 | [0.05, 4.04] | |  |  |  |  |  |  |
| **Radiation therapy (XRT)** |  |  |  |  | 0.101 |  |  |  |  |  |
| XRT | 893 | 1.76 | [0.90, 3.44] | |  |  |  |  |  |  |
| No XRT | 579 | 1.00 | - | |  |  |  |  |  |  |
| **Chemotherapy status** |  |  |  |  | 0.655 |  |  |  |  |  |
| Yes | 957 | 1.16 | [0.61, 2.20] | |  |  |  |  |  |  |
| No | 515 | 1.00 | - | |  |  |  |  |  |  |

***a*** As per National Cancer Institute Surveillance, Epidemiology, and End Results-Medicare requirements, cells containing <11 individuals and any cells making them calculable have been sensored.

***b*** Region groupings are as follows: Northeast (Conneticut and New Jersey); South (Atlanta, rural Georgia, Kentucky, and Louisiana); Midwest (Detroit and Iowa); West (Hawaii, New Mexico, Seattle, Utah and California).

***c*** Urban/rural setting definitions are: Large metro=counties in Metro areas of >/= 1,000,000 population; Metro=counties in metro areas of 250,000 to 1,000,000 population; Urban=urban poplulation >/= 20,000 adjacent or nonadjacent to a metro area; Less urban/rural or rural population of <20,000.
